# "The Hospital's" Nursery Rhymes

**Published:** 1887-04-30

**Authors:** 


### "THE HOSPITAL'S" NURSERY RHYMES.

For Children of all Ages.

### MISS DOYLE AND HER CASTOR OIL.

A very small damsel named Doyle,
Who hated to take castor oil,
Used to say to her mother,
"Give it all to wee brother,
It's certain *my* stomach to spoil."

### THE DUTCH DOCTOR.

A Dutch doctor—old Jacob Van Noogar,—
Always mixed up his medicines with sugar.
All his patients' "small fry"
Hoped he never would die,
This jolly old Doctor Van Noogar.

### "I CAN'T EAT IT."

Mrs. Namby, when seated at table,
Would profess she "to eat was unable."
Mr. Namby said, "Jane,
If from food you abstain
You'll die—and I'll wed cousin Mabel."

### MASTER ROY AND HIS POWDER.

Master Roy, whose full name was Roy Lowder,
Always howled when they gave him a powder;
His mother said, "Roy,
I shall whip you, my boy,
If you make such a fuss for a powder."

